# Probucol inhibits the initiation of atherosclerosis in cholesterol-fed rabbits

**DOI:** 10.1186/1476-511X-12-166

**Published:** 2013-11-04

**Authors:** Manabu Niimi, Yuka Keyamura, Masanori Nozako, Takashi Koyama, Masayuki Kohashi, Reiko Yasufuku, Tomohiro Yoshikawa, Jianglin Fan

**Affiliations:** 1 Department of Molecular Pathology, Interdisciplinary Graduate School of Medicine and Engineering, University of Yamanashi, Yamanashi, Japan; 2 Free Radical Research Project, Otsuka Pharmaceutical Co., Ltd., Tokushima, Japan

**Keywords:** Atherosclerosis, Monocyte, Probucol, Atorvastatin, Hypercholesterolemia

## Abstract

**Background:**

Probucol and statin are often prescribed for treating atherosclerosis. These two drugs exhibit different mechanisms but it is unknown whether they have the same anti-atherogenic properties. In the current study, we examined whether these two drugs at optimal doses could inhibit the initiation of atherosclerosis in cholesterol-fed rabbits in the same way.

**Methods:**

New Zealand White rabbits were fed a cholesterol-rich diet for 5 weeks to produce the early-stage lesions of atherosclerosis. Drug-treated rabbits were administered either probucol or atorvastatin and serum lipids and aortic atherosclerotic lesions were compared with those in a control group.

**Results:**

Atorvastatin treatment significantly reduced serum total cholesterol levels while probucol treatment led to significant reduction of high-density lipoprotein cholesterol levels without changing total cholesterol levels compared with those in the control group. Compared with the control, probucol treatment led to 65% (p < 0.01) reduction while atorvastatin treatment led to 23% (p = 0.426) reduction of the aortic lesion area. Histological and immunohistochemical analyses revealed that the lesions of the probucol-treated group were characterized by remarkable reduction of monocyte adherence to endothelial cells and macrophage accumulation in the intima compared with those of both atorvastatin and control groups. Furthermore, low-density lipoprotein (LDL) isolated from the probucol group exhibited prominent anti-oxidative reaction, which was not present in LDL isolated from either the atorvastatin-treated or the control group.

**Conclusions:**

This study suggests that probucol inhibits the initiation of atherosclerosis by reducing monocyte adherence and infiltration into the subintima. Anti-oxidization of LDL by probucol protects more effectively against early-stage lesion formation than statin-mediated lipid-lowering effects.

## Background

Atherosclerosis is a chronic inflammatory disease that is initiated by monocyte adhesion to arterial intimal surfaces followed by migration into the subintimal space. Intimal monocytes are further differentiated into macrophages and then transformed into foam cells after ingesting deposited lipids. High levels of plasma low-density lipoprotein (LDL), especially oxidized LDL, are the major atherogenic factor for the recruitment of monocyte adhesion and induction of foam cell formation [1,2]. Therefore, reducing plasma LDL levels and inhibition of LDL oxidization are the primary targets for the prevention and treatment of atherosclerotic disease.

Probucol is not only a lipid-lowering drug but also has anti-oxidant activity [3]. For this reason, it is often prescribed for the treatment of hypercholesterolemia and xanthoma. Probucol reduces plasma LDL-cholesterol through increasing LDL catabolism and bile synthesis in the liver [4,5]. However, in addition to LDL-cholesterol lowering effects, probucol also simultaneously reduces plasma high-density lipoprotein (HDL) levels. In the past, low levels of plasma HDL-cholesterol (HDL-C) induced by probucol were considered as a notorious side effect, but now it is clear that the functions of HDL particles such as reverse cholesterol transport [6-8] and scavenger receptor class B type I activity are actually up-regulated [9,10]. Previous studies showed that probucol treatment protects against atherosclerosis in WHHL and cholesterol-fed rabbits [11,12]. Clinically, probucol has been widely used in Asia, such as Japan, China, and Korea, for the treatment of hyperlipidemia and atherosclerosis. Recently, a clinical trial demonstrated that long-term probucol treatment reduced the risk of cardiovascular events in familial hypercholesterolemia [13]. In spite of this, the lipid-lowering effect of probucol is rather moderate compared with that of statins, hydroxymethylglutaryl (HMG)-CoA reductase inhibitors, the most popular lipid-lowering drugs for the treatment of hyperlipidemia. Statins reduce cholesterol biosynthesis through inhibiting HMG-CoA reductase activity and increase hepatic LDL receptor activity [14]. Furthermore, statins also have anti-inflammatory effects designated as pleiotropic effects. Nevertheless, treatment with statin therapy alone can lead to only 30% reduction of the incidence of coronary events [15,16]. Therefore, other non-statin therapies are required for many patients who are not responsive to statins [2,17].

In the current study, we compared these two drugs in terms of atheroprotective effects in cholesterol-fed rabbits. We were particularly interested in the initiation of atherosclerosis and made a side-to-side comparison of these two drugs. Our results suggested that the anti-oxidative effects exerted by probucol may be more beneficial for the inhibition of monocyte adhesion and infiltration during atherogenesis than statin-mediated cholesterol lowering effects.

## Results

### Serum lipids and lipoprotein profile

**Figure 1:**
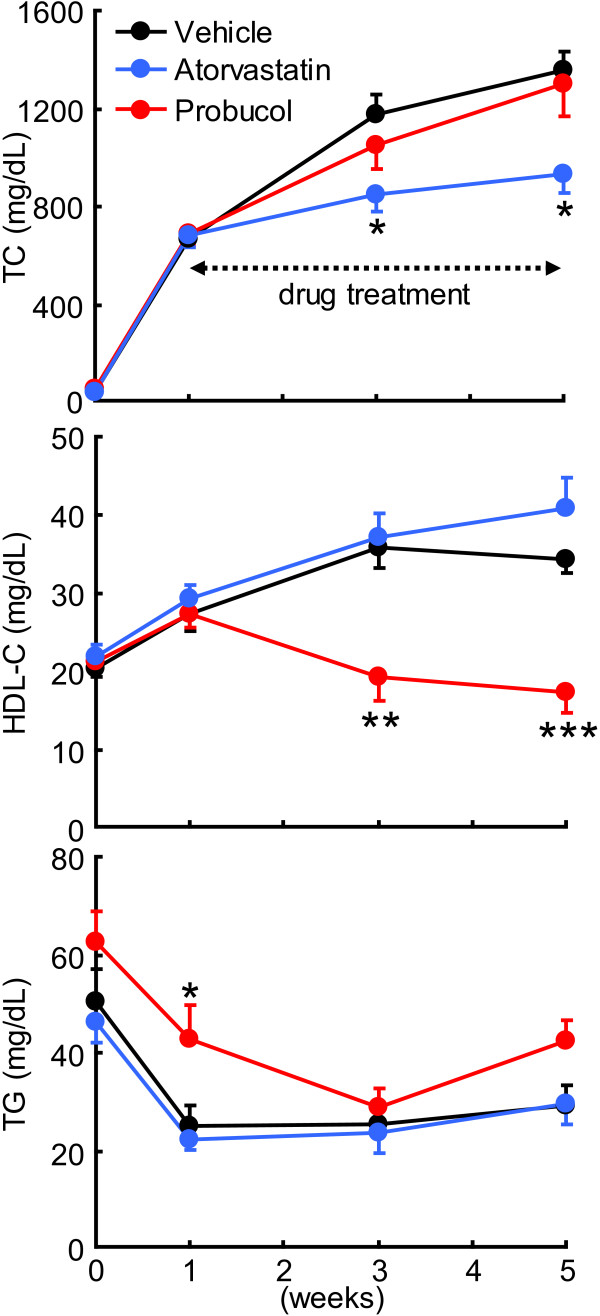
**Serum concentrations of total cholesterol (TC), HDL-cholesterol (HDL-C), and triglycerides (TG).** Data are expressed as mean ± SEM (n = 8, each group). *P < 0.05, **P < 0.01, ***P < 0.001 vs. vehicle.

**Figure 2:**
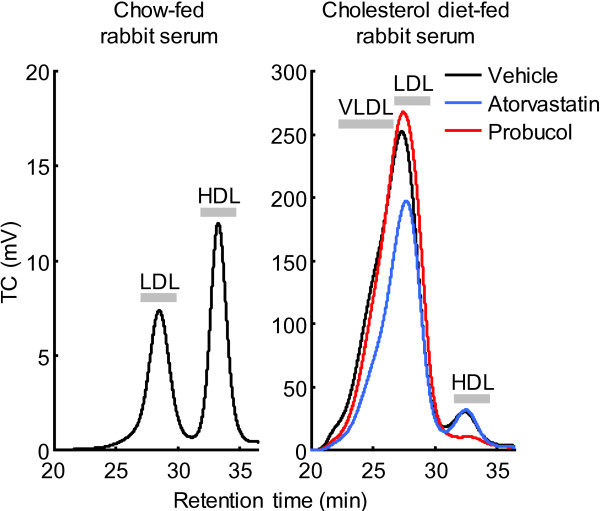
**Analysis of serum lipoprotein profile by HPLC gel filtration.** Serum lipoproteins were fractionated using a gel filtration column with on-line post-column cholesterol detection. Serum lipoprotein profiles of normal rabbits fed a chow diet and cholesterol-enriched for 5 weeks after drug treatment for 4 weeks. Data are expressed as mean value of 8 rabbits from each group.

As shown in Figure 1, the average total cholesterol (TC) level at the baseline (1 week) was 700 mg/dL after cholesterol diet feeding for 1 week. Serum TC level of the vehicle group was increased to 1500 mg/dL at 5 weeks. Atorvastatin treatment led to a significant reduction of serum TC level at 3 and 5 weeks but did not change HDL-C and triglyceride (TG) levels. Probucol treatment did not affect TC and TG levels but significantly reduced HDL-C level at 3 and 5 weeks. Analysis of lipoprotein profiles by high performance liquid chromatography (HPLC) revealed that the increased serum TC level was essentially caused by elevated serum very-low-density lipoprotein and LDL fractions in cholesterol-fed rabbits and these fractions were markedly reduced in the atorvastatin-treated group while HDL fractions were reduced in the probucol-treated group (Figure 2).

### Aortic atherosclerosis

**Figure 3:**
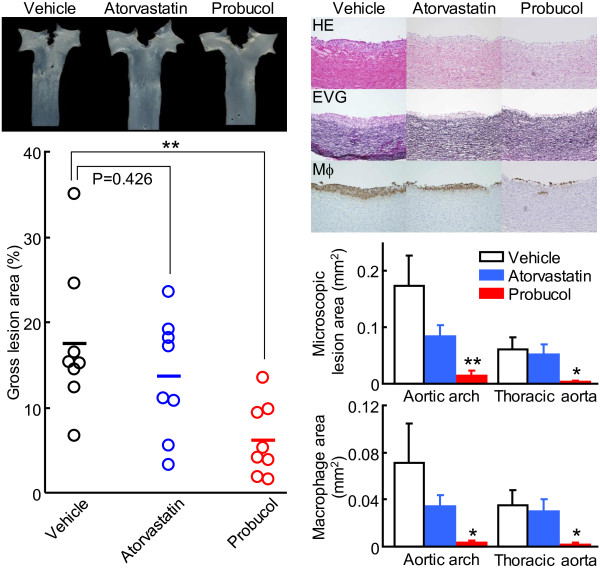
**Analysis of aortic atherosclerosis.** Representative photographs of pinned-out aortic arch from each group (left, top). Scatter plot of the aortic atherosclerotic lesions on the surface was quantified using an image analysis system (left, bottom). Representative micrographs of the aortic arch from each group (right, top). Serial frozen sections of aortas were stained with hematoxylin-eosin (HE), elastica van Gieson (EVG), or immunohistochemically stained with mAb against macrophages (Mϕ). Intimal microscopic lesions on EVG-stained sections (right, middle) and Mϕ-positive area (right, bottom) were quantified with an image analysis system. The values are expressed as mean ± SEM (n = 8, each group). *P < 0.05, **P < 0.01 vs. vehicle.

Analysis of *en face* aortic lesion area revealed that, although both drug treatments inhibited the lesion size (65%↓ in the probucol group and 23%↓ in the atorvastatin group compared with that in the vehicle group), statistical significance was only found in probucol-treated group (Figure 3, left). Histological examinations showed that the aortic lesions were mainly composed of infiltrating macrophages (Figure 3, right). Compared with the vehicle group, intimal lesion area was remarkably reduced in the probucol group (92%↓ in aortic arch and 95%↓ in thoracic aorta). The atorvastatin group also had less intimal lesions, but the difference was not significant (51%↓ in aortic arch and 16%↓ in thoracic aorta, p > 0.05). Immunohistochemical staining showed that the reduced lesion areas found in both drug-treated groups were mainly caused by reduced macrophage infiltration: 96%↓ in aortic arch and 94%↓ in thoracic aorta in the probucol group, and 51%↓ in aortic arch and 15%↓ in thoracic aorta in the atorvastatin group compared with those in the control group, while a significant difference was only found in the probucol group.

### Scanning electron microscopic observation

**Figure 4:**
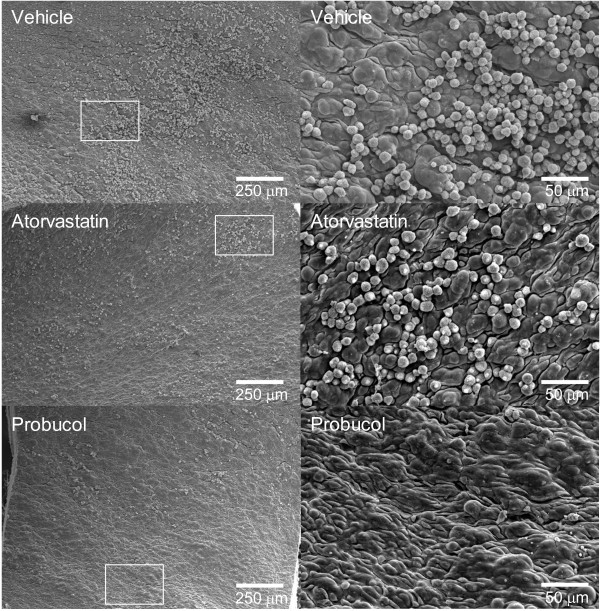
**Representative micrographs of the aortic arch from each group observed under a scanning electron microscope.** At low magnification, monocytes that adhered to the intimal surface of the aorta in the probucol group are small in number compared with those in the atorvastatin and control groups (left). Many monocytes are clearly visible in the atorvastatin and control groups at high magnification (right).

**Figure 5:**
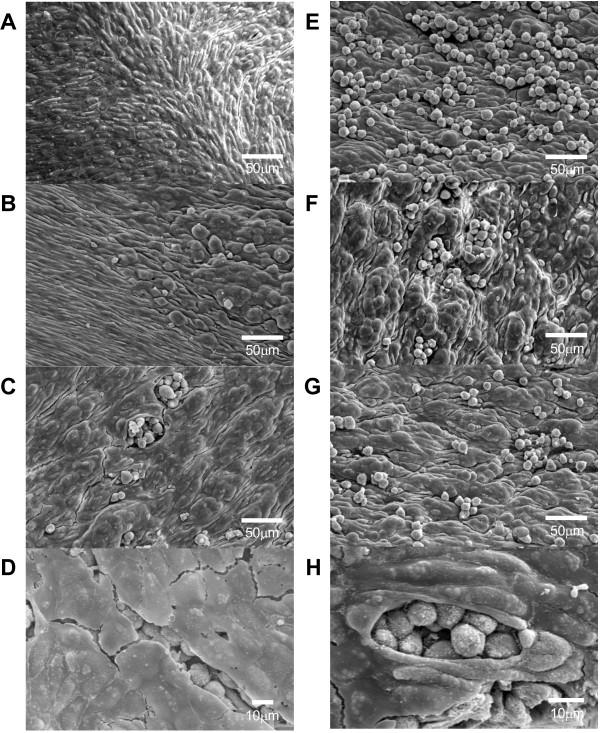
**Different intimal changes of the aorta often observed under a scanning electron microscope. (A)** Normal-appearance intimal endothelial cells. **(B)** Intimal surface becomes bulged without monocyte adherence (right area). **(C-D)** Monocytes are found under split endothelial cells, possibly due to artifacts of the fixation and drying processes. **(E-H)** Monocyte adherence to the bulged or protruded intima.

We also examined the aortic surface changes by scanning electron microscopy and observed monocyte-endothelial interactions. Under the scanning electron microscopy, the lesions of the aorta were visible even at low magnification. While there was variation in lesion features from place to place in the three groups, the number of monocytes adhered to endothelial cells was generally less in the probucol group than in the atorvastatin or control group (Figure 4), which was consistent with the observations in light microscopic histological study described above. At high magnification, there were diverse features of intimal lesions (Figure 5): some areas appeared as raised ridges or became bulged or protuberant with or without monocytes attached on the surface. Many monocytes adhered to the endothelial surface either singly or in clusters. The bulged or protuberant areas contained many monocytes beneath endothelial cells, which can be observed in the areas where endothelial cells were detached or split. These lesions were also fewer in the probucol-treated group than in the other two groups.

### LDL oxidizability

**Figure 6:**
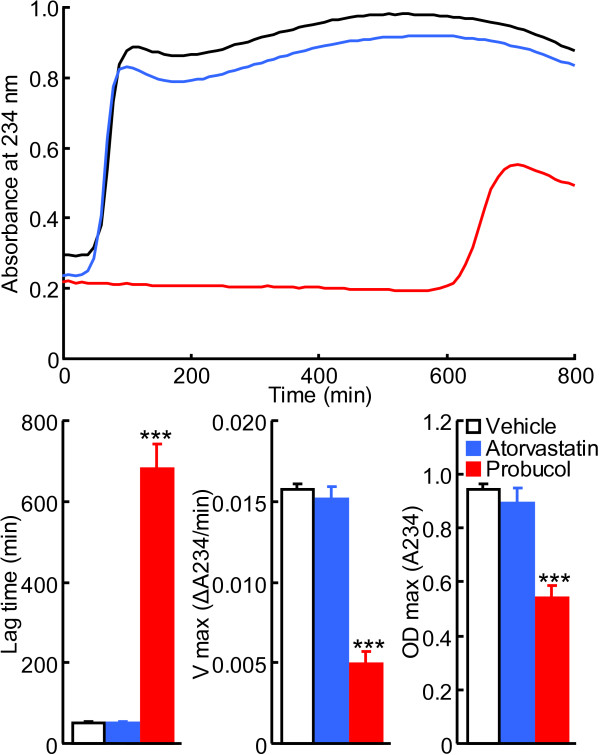
**Kinetic evaluation of LDL oxidation induced by copper.** Representative kinetic graph is shown in the top panel. Lag-time, maximal oxidization speed (V max), and maximal diene concentration (OD max) were calculated. The values are expressed as mean ± SEM (n = 8-9, each group). ***P < 0.001 vs. vehicle.

We compared the suppressive capacity of probucol and atorvastatin on plasma LDL oxidization, a major insult and trigger for atherogenesis. As shown in Figure 6, LDL isolated from probucol-treated rabbits but not atorvastatin-treated rabbits showed significant suppressive ability against copper-induced oxidization expressed as a prolonged lag-time, and decreased maximal oxidation speed and maximal diene concentrations.

## Discussion

In the current study, we compared the atheroprotective effects of probucol and atorvastatin, two hypolipidemic drugs for the treatment of hyperlipidemia. It is generally believed that probucol reduces plasma cholesterol through increasing LDL catabolism and bile excretion, whereas atorvastatin inhibits HMG-CoA reductase activity and increases hepatic LDL receptor activity [14]. Here, we demonstrated that, at the optimal doses of each drug, probucol exerts a greater inhibitory effect on the initiation of cholesterol-diet-induced atherosclerosis in rabbits than atorvastatin. It has been reported previously that probucol at high dose (1%) and for a long period (2 ~ 6 months) protects against atherosclerosis in both WHHL and cholesterol-fed rabbits [11,12]. Here, we further demonstrated that probucol even at lower dose (0.3%) for a short period (4 weeks) was still able to reduce the lesions of atherosclerosis in cholesterol-fed rabbits and the lesions were characterized by reduced intimal macrophage accumulation. It is interesting to note that, unlike atorvastatin, probucol administration for a short period did not lead to a significant cholesterol-lowering effect, while HDL-C was significantly low. Therefore, several possible mechanisms may operate independently upon the lipid-lowering effect regarding probucol’s anti-atherogenic effects. First, probucol has very strong anti-oxidative effects, whereas atorvastatin does not. In this situation, one may consider that the anti-atherogenic effects induced by anti-oxidation by probucol are stronger than the lipid-lowering effects of atorvastatin. It may also suggest that oxidation in the arterial wall may be more important than merely elevation of plasma cholesterol in the initiation of atherosclerosis. The second possible mechanism may be attributed to HDL modulation induced by probucol. It has been reported that HDL functions such as reverse cholesterol transport [6-8] and anti-oxidative activity [18] are markedly up-regulated in probucol-treated patients, while HDL-C levels are low. If these functions occur in the arterial wall, accumulation of lipids would be diminished. Nevertheless, these results may raise an important question: Do we need to treat hyperlipidemic patients with atorvastatin combined with probucol? In Japan, probucol is used for hyperlipidemic patients who are resistant to statins [19,20]. In China, however, probucol is widely used for the treatment of hyperlipidemia and given the same priority as statins. So-called “PAS” therapy (probucol, aspirin, and statin) in China is often prescribed for hyperlipidemic patients for both primary and secondary prevention of coronary artery diseases [21]. Although this question still awaits more clinical investigation, apparently, the anti-oxidative capacity is superior to the lipid-lowering effect for protection against atherosclerosis initiation.

## Conclusions

Our studies provide evidence that probucol inhibited the initiation of atherosclerosis without improving serum TC levels, despite a lowering of serum HDL-C levels. These results suggest that the anti-oxidative effects exerted by probucol are more efficient and effective for the inhibition of atherosclerosis than the cholesterol-lowering effects.

## Methods

### Chemicals

Probucol was synthesized at Otsuka Pharmaceutical Co., Ltd. (Tokushima, Japan). Atorvastatin calcium was purchased from Sequoia Research Products Ltd. (Pangbourne, UK).

### Experimental protocol

Male New Zealand White rabbits (1.0-1.5 kg at age of 6-7 weeks old) provided by Kitayama Labes Co., Ltd. (Nagano, Japan) were fed an atherogenic diet containing 0.5% cholesterol for 1 week. Using the TC levels (around 700 mg/dl), we selected those rabbits that showed a reasonable response to cholesterol diet feeding for the following experiments. These rabbits were further fed with either the same atherogenic diet (vehicle group) or supplemented with either 0.3% (w/w) probucol or 0.005% (w/w) atorvastatin for another 4 weeks. All diets were prepared by Oriental Yeast Co., Ltd. (Tokyo, Japan). Blood samples were obtained from the auricular vein biweekly for biochemical analysis. At the end of the experiment, all rabbits were sacrificed for evaluation of aortic atherosclerosis. All animal experiments were performed with the approval of the Animal Care Committee of Otsuka Pharmaceutical Company and the University of Yamanashi.

### Analysis of serum biochemistry

Serum levels of TC, TG, and HDL-C were measured using commercial assay kits (Wako Pure Chemical Industries Ltd., Osaka, Japan) and automated clinical analyzer BIOLIS 24i (Tokyo Boeki Machinery, Ltd., Tokyo, Japan).

### Analysis of lipoproteins using HPLC

Serum lipoproteins were analyzed using HPLC (Tosoh Co., Tokyo, Japan) according to the procedure described by Usui *et al.*[22] with slight modification. In brief, sera were separated on 2 tandem connected gel filtration columns TSKgel LipopropakXL (Tosoh Co., Tokyo, Japan) with phosphate-buffered saline containing 0.005% Brij-35 and 0.05% sodium azide. The column effluent was mixed with on-line connected cholesterol colorimetric reagent (Wako Pure Chemical Industries Ltd., Osaka, Japan) and the absorbance at 600 nm was monitored after the enzymatic reaction in a reaction coil at 37°C. The lipoprotein fractions of very-low-density lipoprotein, LDL, and HDL were defined based on retention time.

### Analysis of aortic atherosclerosis

At the end of the experiment, all rabbits were euthanized by injection of an overdose of sodium pentobarbital solution. Aortas were rapidly dissected free from the arch to the iliac artery bifurcation. The aortas were analyzed for *en face* gross lesions and microscopic quantification of intimal lesion areas. *En face* lesion area was quantitated as described previously [23]. Aortas were opened longitudinally and mounted on a black rubber board, and then were photographed using a digital camera. Using Photoshop software (Adobe Systems Inc., San Jose, CA), atherosclerotic lesions were visualized first and then quantitatively measured using the image analysis system WinRoof (Mitani Co., Tokyo, Japan). For microscopic lesion analysis, two segments (aortic arch and thoracic aorta) fixed in 10% formalin were embedded in OCT compound (Sakura Finetek Japan Co., Ltd., Tokyo, Japan). Frozen sections were stained with hematoxylin-eosin (HE) and elastica van Gieson (EVG). The intimal lesion size was quantitatively measured using the image analysis system WinRoof as described previously [24]. For microscopic evaluation of the cellular components of the lesions, serial frozen sections of the aorta were immunohistochemically stained with RAM11 (Dako Japan Inc., Tokyo, Japan) monoclonal antibody against rabbit macrophage using Histofine Simple Stain MAX-PO(M) kits (Nichirei Biosciences Inc., Tokyo, Japan). Macrophages in the lesions were measured by quantifying the positively stained area using the image analysis system.

### Scanning electron microscopic observation

To observe intimal surface changes of aorta after cholesterol-rich diet feeding, we perfused three rabbits from each group at the end of the experiment using phosphate-buffered saline followed by fixative solution containing 2.5% glutaraldehyde and 2% paraformaldehyde (pH 7.4) for 20 min. After that, aortas were dissected free from the aortic root to the common carotid artery bifurcation. Aorta was cut into 13 pieces as specimens (approximately 3 mm square). These specimens were further fixed in the same solution at 4°C overnight. Fixed specimens were post-fixed with 1% osmium tetroxide (pH 7.4) and dehydrated with graded ethanol followed by isoamyl acetate. Aortic segments were dried in CO_2_ using critical point dryer HCP-2 (Hitachi Koki Co. Ltd., Tokyo, Japan) and were sputter-coated with a thin gold layer in an ion coater IB-3 (EIKO Co., Tokyo, Japan). The aortic surface was examined under a scanning electron microscope JSM-6510 (JEOL Ltd., Tokyo, Japan).

### Evaluation of LDL oxidizability

Plasma LDLs (d = 1.006-1.063) were isolated by ultracentrifugation [25] from rabbits at 4 weeks after cholesterol-rich-diet feeding. The susceptibility of LDL to copper-induced oxidation was assessed by determining the formation of conjugated dienes as described previously [26]. In brief, LDL (50 μg protein/mL) was incubated with 5 μM CuSO_4_, and the formation of conjugated dienes was determined by monitoring the change of absorbance at 234 nm at 37°C with a microplate reader SpectraMax 190 (Molecular Devices, Sunnyvale, CA). Lag-time, maximal oxidization speed, and maximal diene concentrations were calculated for the estimation of oxidization sensitivity.

### Statistical analysis

All data were expressed as mean ± SEM. Statistical significance was determined by one-way ANOVA (Dunnett’s test) using SAS software (SAS Institute Japan Ltd., Tokyo, Japan). P values less than 0.05 were considered significant or otherwise specified.

## Competing interests

The authors declare no conflict of interest.

## Authors’ contributions

MN, TY and JF designed the study. MN, YK, MN, TK, MK and RY performed the experiments and MN analyzed the data. MN and JF wrote the manuscript. All authors read and approved the final manuscript.
